# Detection of Novel Poxvirus from Gray Seal (*Halichoerus grypus*), Germany

**DOI:** 10.3201/eid2906.221817

**Published:** 2023-06

**Authors:** Florian Pfaff, Katharina Kramer, Jacqueline King, Kati Franzke, Tanja Rosenberger, Dirk Höper, Patricia König, Donata Hoffmann, Martin Beer

**Affiliations:** Friedrich-Loeffler-Institute, Greifswald, Germany (F. Pfaff, J. King, K. Franzke, D. Höper, P. König, D. Hoffmann, M. Beer);; Landeslabor Schleswig-Holstein, Neumünster, Germany (K. Kramer);; Seehundstation Friedrichskoog e.V., Friedrichskoog, Germany (T. Rosenberger)

**Keywords:** poxvirus, viruses, zoonoses, poxviridae, Chordopoxvirinae, seals, North Sea, sequencing, phylogeny, genome, Germany, Halichoerus grypus, gray seal, Wadden Sea poxvirus

## Abstract

We detected a novel poxvirus from a gray seal (*Halichoerus grypus*) from the North Sea, Germany. The juvenile animal showed pox-like lesions and deteriorating overall health condition and was finally euthanized. Histology, electron microscopy, sequencing, and PCR confirmed a previously undescribed poxvirus of the *Chordopoxvirinae* subfamily, tentatively named Wadden Sea poxvirus.

Members of the poxvirus subfamily *Chordopoxvirinae* (family *Poxviridae*) infect vertebrates, such as birds, reptiles, and a broad spectrum of mammals. Although some chordopoxviruses have a narrow host range, several can easily jump species barriers and cause severe disease (*1*). Considering the potential zoonotic and epizootic potential of chordopoxviruses, constant monitoring and adaptation of diagnostic procedures are essential. With the advent of metagenomic sequencing, novel chordopoxviruses have been identified that are genetically diverse and were not readily detectable by using established PCR-based diagnostics (*2*–*4*).

We report a case of a poxvirus infection in a gray seal (*Halichoerus grypus*) from the North Sea near Germany. We identified a novel chordopoxvirus that was phylogenetically divergent from other known poxviruses of gray seals.

## Case Study

**Figure 1:**
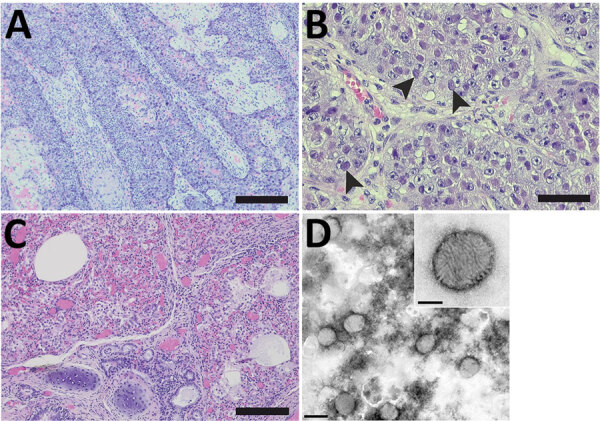
Histopathology and electron microscopy of nodules and lung tissue from a gray seal (*Halichoerus grypus*) with novel poxvirus, North Sea, Germany. A) Histopathology of nodules shows severe papillary epithelial hyperplasia with infiltration of neutrophils. Scale bar indicates 200 μm. B) Histopathology of ballooning degeneration of epithelial cells. Arrows indicate large eosinophilic intracytoplasmic inclusion bodies. Scale bar indicates 50 μm. C) Histopathology of the lung shows multifocal moderate pneumonia with infiltration of mononuclear cells and neutrophils with proliferation of pneumocytes type II and intra-alveolar histiocytosis, severe atelectasis, and hyperemia. Scale bar indicates 100 μm. D) Negative-contrast electron microscopy of lung tissue. Microscopy revealed poxvirus-like viral particles. Scale bar indicates 300 nm. Inset: closeup of poxvirus-like particles, which had an oval shape ≈250 nm × ≈200 nm and an irregular surface with randomly arranged tubular structures; scale bar indicates 100 nm.

In June 2020, a juvenile gray seal was nursed at a rehabilitation center in Friedrichskoog, Germany (Appendix Figure 1) and was about to be released back into the wild when staff noticed pox-like lesions on its hind flipper (Appendix Figure 2). The seal’s overall health condition deteriorated over the next 3 weeks, and it had dyspnea, vomiting after feeding, and apathy; it was humanely euthanized. At necropsy, the seal was in good body condition (abdominal blubber ≈35 mm, reference >30 mm). We noted 2 prominent verrucous nodules on the right hind flipper (Appendix Figure 2). We also found severe emphysema of the mediastinum and a focal, adhesive pleuritis. No other organs had lesions. Histologic examination of both nodules (Appendix) revealed severe papillary epithelial hyperplasia, acanthosis, ballooning degeneration, large eosinophilic cytoplasmatic inclusion bodies in the stratum spinosum, moderate hyperkeratosis, and severe ulceration with hemorrhages (Figure 1, panels A, B). In addition, we observed multifocal severe infiltrations of neutrophils. In the liver, we detected a focally necrotizing hepatitis with ballooning degeneration of nuclei and a focal granulomatous subcapsular hepatitis with intralesional parasites and calcification. We found further inflammatory changes in the lungs (Figure 1, panel C), which had multifocal moderate pneumonia with infiltration of mononuclear cells and neutrophils; the heart had focal severe mononuclear myocarditis; and the duodenum had moderate diffuse lymphoplasmacellular enteritis. We also observed depletion of lymphocytic organs, including a severe atrophy of the thymus.

Results of quantitative PCRs (qPCRs) specific for orthopoxvirus (*5*) and parapoxvirus (*6*), canine alphaherpesvirus 1, influenza A virus, canine morbillivirus, and *Brucella* sp. were negative for lung and skin lesion tissue. We isolated an *Escherichia coli* strain from lung, mediastinum, liver, kidney, and intestines.

We used electron microscopy to analyze lung tissue and detected typical poxvirus-like virions, which were ovoid in shape and ≈250 nm long and ≈200 nm wide (Figure 1, panel D). The surface structures resembled typical orthopox-like randomly arranged tubular units. However, the virion morphology did not enable assignment to a poxvirus genus.

We isolated DNA from a pool of lung and skin lesion tissue and sequenced DNA using Ion Torrent S5XL (Thermo Fisher Scientific, https://www.thermofisher.com) (*7*), NovaSeq (Illumina, https://www.illumina.com), and MinION Mk1C (Oxford Nanopore Technologies, https://nanoporetech.com) sequencing technologies (Appendix). We combined the reads in a hybrid assembly, which resulted in a complete poxviral genome (mean coverage ≈650). We were able to confirm completeness of the genome because the terminal repeats contained the terminal hairpin region.

We screened several organs by using 2 different virus-specific qPCRs (Appendix). Results from both qPCR panels were consistent and we detected the highest viral loads in the skin lesion and parts of the lung (Table).

**Table:** Quantitative PCR detection of novel poxvirus from different tissues of a gray seal (*Halichoerus grypus*), Germany*

Sample	Cycle quantification value	
	Panel 1, viral DNA polymerase	Panel 2, viral RNA polymerase
Skin lesion	9.1	8.9
Lung 1	18.2	18.2
Lung 2	32.3	31.3
Lymph nodes	25.0	24.6
Uterus	26.5	26.2
Spleen	27.2	27.2
Kidney	28.8	28.8
Blood, EDTA	32.2	31.5
Brain	32.9	33.7
Liver	33.7	32.7

*Novel virus is tentatively named Wadden Sea virus.

We tentatively named the poxvirus Wadden Sea poxvirus (WSPV) to reflect the geographic origin of the infected gray seal, which was found in the Wadden Sea, an intertidal zone in the southeastern part of the North Sea, Germany (Appendix Figure 1). We submitted the annotated WSPV genome sequence to the International Nucleotide Sequence Database Collaboration (https://www.insdc.org; accession no. OP810554).

WSPV had one of the smallest genomes (124,614 bp) and lowest guanine-cytosine content (≈22.5%) described so far among chordopoxviruses. The unique core genome of 117,842 bp was flanked by 2 inverted terminal repeats of 3,386 bp each. We identified 124 unique potential open reading frames (ORFs), of which 3 were duplicated in the inverted terminal repeats. BLASTp (https://blast.ncbi.nlm.nih.gov/Blast.cgi) identified 111 ORFs representing orthologs of poxvirus proteins. Nine ORFs encoded proteins that were not related to known poxvirus proteins but showed sequence similarity to eukaryote proteins, and 4 ORFs remained unclassified.

**Figure 2:**
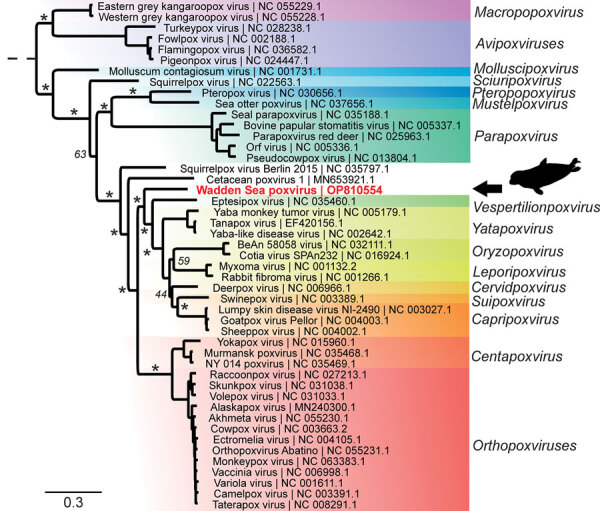
Phylogenetic tree of novel poxvirus detected from gray seal (*Halichoerus grypus*), Germany. Sequencing resulted in a complete poxvirus genome and the virus was tentatively named Wadden Sea poxvirus (red text). Phylogenetic analysis of 15 concatenated viral proteins (alignment of 9,130 aa) showed that Wadden Sea poxvirus (black arrow) is a member of the subfamily *Chordopoxvirinae* but might resemble a novel species distant from the established genera. Asterisks indicate major branches of the bootstrap support at >90%. Scale bar indicates amino acid substitutions per site.

For phylogenetic classification, we compared the amino acid sequences encoded by 15 poxvirus core genes from WSPV with the respective homologs from 47 representative poxviruses (Appendix). WSPV formed a separate phylogenetic branch that did not fall within any of the established genera (Figure 2) and likely is a new species within a novel genus of the subfamily *Chordopoxvirinae*. Of note, a sequence comparison of the predicted WSPV DNA polymerase protein to the nonredundant BLAST database revealed a 96.3% sequence identity with a partial sequence from a Steller sea lion poxvirus (GenBank accession no. AAR06586.1), but other poxviruses had a sequence identity <77%.

## Conclusions

Infections with poxviruses have been reported from gray seals and harbor seals (*Phoca vitulina*), both of which live in the North Sea, Germany. Poxviruses have also been reported in other pinniped species and infections are usually associated to subclades of parapoxviruses, called sealpox or sea lion pox virus (*8*–*10*). Ulcerative to proliferative, nodular, cutaneous, and mucosal lesions have been found in seals infected with parapoxviruses (*10*–*12*). The nodules usually heal spontaneously, but healing lasts from several weeks up to a few months. The illness rate is high, but death rates are low (*13*). Rarely, nodules in the oral cavity can lead to problems during feeding, and secondary bacterial infection can fatally impair respiratory functions (*14*). However, in the case we report, we could not link the animal’s overall deteriorating health condition and severe pneumonia to oral lesions. We considered the cultured *E. coli* strain a facultative pathogen that might have been involved in disease; however, postmortem contamination might be more likely.

We did not detect any parapoxvirus DNA, but sequencing revealed WSPV, a novel poxvirus that is phylogenetically distinct from any other members of the subfamily *Chordopoxvirinae*. The high loads of viral DNA in several organs (Table), and the observed histopathologic changes suggested a generalized infection with systemic pathology. Lesion associated detection of high viral loads (cycle quantification [cq] values for skin cq ≈9, for lung cq ≈18) indicated that the WSPV infection was likely responsible for the gray seal’s disease and severe pneumonia. However, other factors might have been involved, and the source of infection, the potential natural reservoir, and the zoonotic potential of WSPV are unknown. None of the contact animals within the rehabilitation center had similar lesions develop and so far, no further cases have been reported.

Sequence comparison of WSPV showed that a close relative of this novel poxvirus has been detected in a cutaneous lesion of a young Steller sea lion (*Eumetopias jubatus*) from Prince William Sound, Alaska, USA (*15*). This finding suggests a geographically wide distribution of WSPV or WSPV-related viruses and the potential to infect other pinnipeds. As noted in the case we describe, WSPV can cause severe disease. Therefore, future diagnostic considerations for pox-like lesions of pinniped species should include WSPV.

Appendix Additional information on novel poxvirus detected from a gray seal (*Halichoerus grypus*), Germany.
