# Supplementary material for: Detection of Novel Poxvirus from Gray Seal (Halichoerus grypus), Germany
Source: Emerg Infect Dis. 2023 Jun;29(6):1202–5. doi: 10.3201/eid2906.221817 (PMC10202864; doi:10.3201/eid2906.221817)
Supplement: Appendix — Additional information on novel poxvirus detected from a gray seal (Halichoerus grypus), Germany. [file 22-1817-Techapp-s1.pdf]

Article DOI: https://doi.org/10.3201/eid2906.221817

EID cannot ensure accessibility for supplementary materials supplied by authors. Readers who have difficulty accessing supplementary content should contact the authors for assistance.

Detection of Novel Poxvirus from Gray Seal (*Halichoerus grypus*), Germany

Appendix

Materials and Methods

Histopathology

For histology, tissue samples of skin, lung, heart, thymus, mediastinum, liver, spleen, kidney, urinary bladder, esophagus, stomach, intestine, pancreas, ovary, bone marrow, brain, and lymph nodes were collected and fixed in 10% non-buffered formalin, processed by routine methods, embedded in paraffin wax, sectioned at 2 µm and stained with hematoxylin and eosin (HE) using standard methods.

Nucleic Acid Extraction

Prior to DNA extraction, ≈50 mg of native organ samples were homogenized using the TissueLyser II (Qiagen, Germany) in combination with 1 mL cell culture medium (mixture of equal volumes of Eagle Minimum Essential Medium (MEM) (Hank's balanced salts solution) and Eagle MEM (Earle's balanced salts solution), 2 mM L-Gln, nonessential amino acids, adjusted to 850 mg/L $NaHCO_3$, 120 mg per L sodium pyruvate, pH 7.2 with 10% FCS (Bio & Sell GmbH, Germany) and a stainless-steel bead.

For metagenomic sequencing, a pool of homogenized lung and skin lesion tissue was centrifuged and the pellet was collected in 1 mL LBE buffer. Total DNA then was extracted using the RNAdvance Tissue kit (Beckman Coulter, Germany) in combination with a KingFisher Flex system (Thermo Fisher Scientific, Germany) skipping the DNase I digestion step.

For screening different organs for presence of viral DNA by qPCR, supernatants from the homogenization step were collected. DNA was extracted using the QIAamp DNA Mini Kit (Qiagen) according to the manufacturer's instructions.

Electron Microscopy

For transmission electron microscopy, tissue from the lung was homogenized and diluted in media. The suspension was used for adhesion to Formvar coated EM grids (Ni, 400 mesh). The EM grids were additional coated with carbon and glow discharged before adhesion to the sample. 1% phosphor tungstic acid pH 6 was used for negative staining and the samples were analyzed by a Talos F200i transmission electron microscope (Thermo Fisher Scientific) at an acceleration voltage of 80 kV. The used detector is the Ceta 16M Camera together with Velox Imaging Software (Thermo Fisher Scientific).

Sequencing

The DNA from a pool of lung and skin lesion tissue was sequenced using 3 different platforms: Ion Torrent, nanopore, and Illumina.

For Ion Torrent, DNA was used for library preparation as described in Wylezich et al. 2018 (*1*). The library was then sequenced using an Ion Torrent S5XL system (Life Technologies, Germany).

For nanopore long read sequencing, DNA was prepared by using an SQK-RBK004 Rapid Barcoding Kit (Oxford Nanopore Technologies, UK) following the manufactures instructions. The final library was run on a MinION Flow Cell (R9.4.1) for 19h 46m using a Mk1C device (Oxford Nanopore Technologies, UK).

For Illumina, DNA was submitted to Eurofins Genomics Germany and sequenced using a NovaSeq instrument (Illumina, USA) running in paired-end mode (2 × 150 bp).

Sequence Analysis

Raw Illumina paired-end reads were trimmed to remove bad quality regions and adaptor contamination using TrimGalore! version 0.6.5. A subset of the trimmed paired-end reads along with the Nanopore long reads were de novo assembled using SPAdes version 3.15.4; (*2*) and Unicycler version 0.4.4, both running in hybrid mode. The resulting contigs were visualized, arranged, and annotated using Geneious Prime version 2021.0.1. Open reading frames were predicted and translated, and subsequently classified using BLASTp version 2.10.1 against a filtered version of the "nr" database that contained only entries belonging to the taxonomic superkingdoms "Viruses" (NCBI:txid10239) and "Eukaryota" (NCBI:txid2759).

Phylogenetic Analysis

The amino acid (aa) sequences of 15 predicted ORFs from Wadden Sea poxvirus (WSPV) were aligned to their respective homologs (Appendix Table 1) from 47 representative poxviruses. Individual amino acid alignments were generated using MUSCLE version 3.8.425 (*3*). The alignments were subsequently concatenated and used for phylogenetic analysis using IQ-TREE2 version 2.2.0 (*4*). In detail, we used a partition model (*-p*) to reflect the nature of the multi gene alignment allowing for individual substitution models and evolutionary rates (*5*). The optimal substitution model for each partition was selected using ModelFinder, *-m MFP* (*6*). Branch support was calculated using 100,000 ultra-fast bootstraps, *-bb 100000* (*7*).

Quantitative PCR

Two sets of specific primer/probe sets (Appendix Table 2) for real-time quantitative PCR were designed using Primer3web version 4.1.0 targeting the viral DNA- and RNA-polymerase genes of the WSPV genome. The AgPath-ID One-Step RT-PCR Kit (Applied Biosystems, Germany) was used according to the manufacturer's instructions and the reaction was performed with the following cycler setup: 95°C for 10 min, 42 cycles of 95°C for 15 sec, 57°C for 20 sec.

References

Wylezich C, Papa A, Beer M, Höper D. A versatile sample processing workflow for metagenomic pathogen detection. Sci Rep. 2018;8:13108. PubMed https://doi.org/10.1038/s41598-018-31496-1

2. Prjibelski A, Antipov D, Meleshko D, Lapidus A, Korobeynikov A. Using SPAdes de novo sssembler. Curr Protoc Bioinformatics. 2020;70:e102. PubMed https://doi.org/10.1002/cpbi.102

3. Edgar RC. MUSCLE: a multiple sequence alignment method with reduced time and space complexity. BMC Bioinformatics. 2004;5:113. PubMed https://doi.org/10.1186/1471-2105-5-113

4. Minh BQ, Schmidt HA, Chernomor O, Schrempf D, Woodhams MD, von Haeseler A, et al. IQ-TREE 2: new models and efficient methods for phylogenetic inference in the genomic era. Mol Biol Evol. 2020;37:1530–4. PubMed https://doi.org/10.1093/molbev/msaa015

5. Chernomor O, von Haeseler A, Minh BQ. Terrace aware data structure for phylogenomic inference from supermatrices. Syst Biol. 2016;65:997–1008. PubMed https://doi.org/10.1093/sysbio/syw037

6. Kalyaanamoorthy S, Minh BQ, Wong TKF, von Haeseler A, Jermiin LS. ModelFinder: fast model selection for accurate phylogenetic estimates. Nat Methods. 2017;14:587–9. PubMed https://doi.org/10.1038/nmeth.4285

7. Hoang DT, Chernomor O, von Haeseler A, Minh BQ, Vinh LS. UFBoot2: improving the ultrafast bootstrap approximation. Mol Biol Evol. 2018;35:518–22. PubMed https://doi.org/10.1093/molbev/msx281

and 72°C for 30 sec on a Bio-Rad CFX96 qPCR cycler (Bio-Rad, Germany).

Appendix Table 1. Protein coding genes used for phylogenetic analysis of novel poxvirus from grey seal, Germany*

Gene	Gene product	Alignment length, aa
J6R	DNA-dependent RNA polymerase subunit RPO147	1,298
A24R	DNA-dependent RNA polymerase subunit RPO132	1,197
D5R	NTPase	819
E8R	Membrane protein E8	805
A3L	Virion major core protein P4b	789
A18R	DNA helicase	714
D6R	Early transcription factor small subunit VETF-s	656
E6R	IMV membrane protein E6	577
D13L	Trimeric virion coat protein	575
A32L	ATPase	367
D12L	mRNA capping enzyme small subunit	314
G8R	Late transcription factor VLTF-1	268
D4R	Uracil-DNA glycosylase	268
L1R	IMV transmembrane protein	258
A2L	Late transcription factor VLTF-3	233
Total		9,138

*Gene nomenclature is analog to that of Vaccinia virus. Novel poxvirus tentatively named Wadden Sea poxvirus. aa, amino acid

Appendix Table 2. Sequences of oligonucleotides and hybridization probes used for specific real-time quantitative PCR detection of novel poxvirus from grey seal, Germany*

Oligo ID	Sequence, 5′→3′	Genome position†	Gene
WSPV_Hyb1	6Fam- AGAAACAAATTGTGTGGATCTGGGTTCACA -BHQ1	29901–29930	DNA polymerase
WSPV_F1	GAATTGTTCCCTCTGTTCGTCT	29861–29882	DNA polymerase
WSPV_R1	AAATACCCACCTCCTCAATACA	29963–29942	DNA polymerase
WSPV_Hyb2	6Fam- CGTGCTATATTGGGATGTGCTCAGGCTAAA -BHQ1	103403–103432	RNA polymerase
WSPV_F2	TGTTGCATCATCATTAGTTGGA	103360–103381	RNA polymerase
WSPV_R2	TCAGAACTAAGACACGATATTGCT	103458–103435	RNA polymerase

*Novel poxvirus tentatively named Wadden Sea poxvirus (WSPV).
†Position based on WSPV reference genome, International Nucleotide Sequence Database Collaboration (https://www.insdc.org) accession no. OP810554.

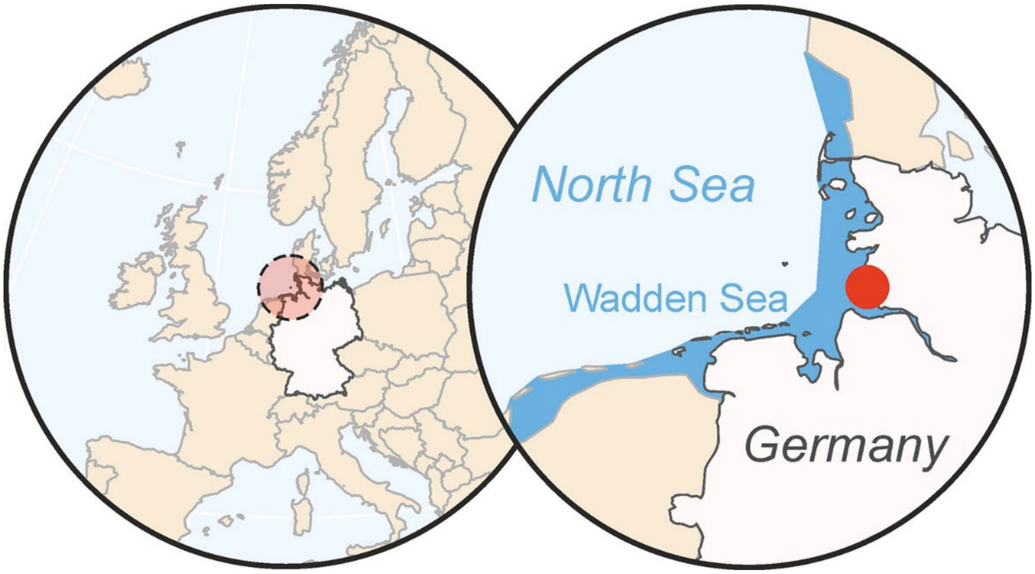

Appendix Figure 1. Map of area where a gray seal (*Halichoerus grypus*) with novel poxvirus was found and nursed, Germany. Map on left shows Germany (white); map on right shows enlarged area of the red circle in the left map and detail of the Waddell Sea area of the North Sea where the seal was found. Red dot indicates location of the rehabilitation facility in northwestern Germany where the gray seal was nursed.

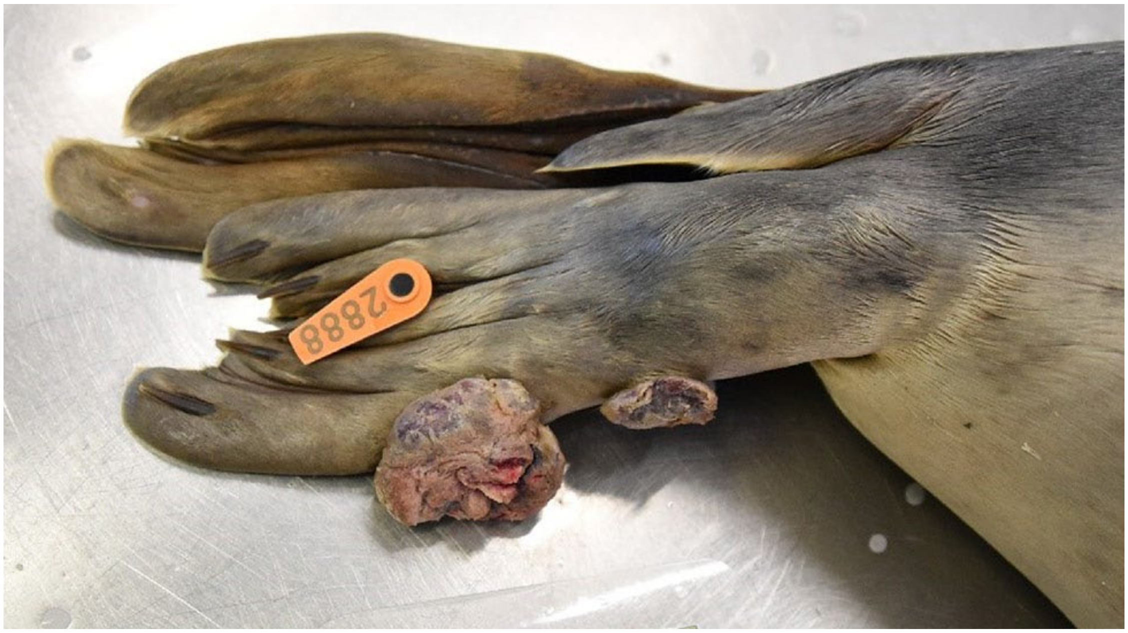

Appendix Figure 2. Verrucous nodules on the right hind flipper of a gray seal (*Halichoerus grypus*) with novel poxvirus, North Sea, Germany. Histopathology of nodules showed severe papillary epithelial hyperplasia with infiltration of neutrophils, ballooning degeneration of epithelial cells, and large eosinophilic intracytoplasmic inclusion bodies (Figure 1).
